# Functional movement compensations persist in individuals with hip osteoarthritis performing the five times sit-to-stand test 1 year after total hip arthroplasty

**DOI:** 10.1186/s13018-020-01663-0

**Published:** 2020-04-16

**Authors:** Anna-Clara Esbjörnsson, Josefine E. Naili

**Affiliations:** 1 Skane University Hospital, Department of Clinical Sciences Lund, Orthopaedics, Lund University, 221 85 Lund, Sweden; 2 Department of Women’s and Children’s Health, Karolinska Institutet, Karolinska University Hospital, Motoriklab, Q2:07, 171 76 Stockholm, Sweden

**Keywords:** Joint replacement, Hip osteoarthritis, Motion analysis, Function, Outcome

## Abstract

**Background:**

Methods to quantify and evaluate function are important for development of specific rehabilitation interventions. This study aimed to evaluate functional movement compensation in individuals with hip osteoarthritis performing the five times sit-to-stand test and change following total hip arthroplasty. To this end, trajectories of the body’s center of mass in the medial-lateral and anterior-posterior dimensions were quantified prior to and 1 year after total hip arthroplasty and compared to a healthy control group.

**Methods:**

Twenty-eight individuals with hip osteoarthritis and 21 matched healthy controls were enrolled in this prospective study. Within 1 month prior to and 1 year after total hip arthroplasty, performance on the five times sit-to-stand test was evaluated using three-dimensional motion analysis and perceived pain using a visual analog scale. The center of mass trajectories for the medial-lateral and the anterior-posterior dimensions were identified, and the area under the curve was calculated, respectively. Repeated measures ANOVA were used to evaluate differences in the area under the curve, between pre- and postoperative performance, and between participants with hip osteoarthritis and controls.

**Results:**

Preoperatively, individuals with hip osteoarthritis displayed a larger contralateral shift (*p* < 0.001) and forward displacement of the center of mass (*p* = 0.022) compared to controls. After surgery, deviations in both dimensions were reduced (medial-lateral *p* = 0.013; anterior-posterior *p* = 0.009). However, as compared to controls, the contralateral shift of the center of mass remained larger (*p* = 0.010), indicative of persistent asymmetric limb loading. Perceived pain was significantly reduced postoperatively (*p* < 0.001).

**Conclusions:**

By quantifying the center of mass trajectory during five times sit-to-stand test performance, functional movement compensations could be detected and evaluated over time. Prior to total hip arthroplasty, individuals with hip osteoarthritis presented with an increased contralateral shift and forward displacement of the center of mass, representing a strategy to reduce pain by unloading the affected hip and reducing required hip and knee extension moments. After surgery, individuals with total hip arthroplasty displayed a persistent increased contralateral shift as compared to controls. This finding has implications for rehabilitation, where more focus must be directed towards normalizing loading of the limbs.

## Introduction

Osteoarthritis (OA) of the hip is characterized by progressive degeneration of the joint [1, 2]. The disease includes deterioration of articular cartilage, synovium, and subchondral bone, resulting in pain and decreased function [1–3]. Muscle weakness, primarily in the hip and knee extensors and flexors, is common in individuals with hip OA, although the body of evidence is not as substantial as for individuals with knee OA [4]. Reported causes of reduced muscle strength include muscle atrophy by pain-mediated disuse, reduced physical activity, and age [5].

It is recommended that physical function in individuals with OA be assessed using both performance-based tests and patient-reported outcomes [6]. The five times sit-to-stand test (5STS) is a valid performance-based test that is associated with lower limb strength and has been suggested a proxy measure of quadriceps strength [7–10]. The test is performed by measuring the time taken to stand up from a seated position five times as fast as possible, which makes the test easy to use in clinical practice. Previous research investigating sit-to-stand performance in individuals with hip OA report functional movement compensations and asymmetries as compared to controls [11–14]. By observing the body’s center of mass (CoM) and its trajectory during a standardized sit-to-stand test, asymmetrical movement may be quantified as a lateral shift of the CoM towards the contralateral (non-affected) limb. Reduced muscle strength, predominantly involving the knee extensors, may manifest as increased forward displacement of the CoM. This has previously been demonstrated in individuals with knee OA, where quantification of the CoM trajectory during the 5STS was shown to be a sensitive and responsive measure prior to and 1 year after total knee arthroplasty (TKA) [15]. The sit-to-stand movement is important as it occurs many times each day and allows an individual to move independently. Older community-dwelling individuals are reported to perform at least 45 sit-to-stand transitions per day [16].

Patient reported outcomes, including evaluation of both pain and function, following joint arthroplasty are more favorable among individuals with THA as compared to individuals with TKA [17–19]. Furthermore, the perception that postoperative rehabilitation following THA is not as crucial as following a TKA exists. However, patients with THA express that recovery following surgery is challenging due to slow progress and reduced physical functioning [20]. Since functional movement compensations are present already in individuals with mild-to-severe hip OA [13, 21], and they seem to persist long after surgery [22, 23], methods to quantify functional movement compensations and evaluate over time are important. This is to enable development of targeted rehabilitation interventions. Therefore, the primary aim of this study was to (1) evaluate whether the CoM trajectory is able to detect functional movement compensations in individuals with hip OA performing the 5STS compared to controls, (2) evaluate change in the CoM trajectory 1 year after THA, and (3) evaluate whether any deviations from normal persists 1 year after THA. In addition, we wanted to explore whether the number of consecutive sit-to-stand cycles had any impact on the CoM trajectory. It was hypothesized that individuals with hip OA would display an increased contralateral shift of the CoM, and an increased forward displacement of the CoM as compared to controls, and that these would be reduced postoperatively as compared to preoperatively.

## Materials and methods

### Study design and participants

A cohort of 40 individuals with physician-diagnosed primary hip OA, and 25 age- and gender-matched controls with no known musculoskeletal disease, were recruited for this prospective study between the years 2011 and 2015 [24]. Inclusion criteria included being scheduled for THA within 1 month after baseline evaluation, ability to walk 10 m repeatedly without the use of a walking aid, and ability to understand written and verbal information in Swedish. Exclusion criteria included previous major orthopedic surgery in the lower limbs, other lower extremity joint pain or severe back pain, rheumatoid arthritis, diabetes mellitus, neurologic disease, BMI > 40, and/or other condition affecting walking ability. Individuals with hip OA were recruited from two orthopedic departments in Stockholm, Sweden (Ortho Center Löwenströmska Hospital and Karolinska University Hospital). The control group was recruited through acquaintances and matched by age to the OA group. All participants provided written and verbal informed consent to participate in the study in accordance with the Declaration of Helsinki. The regional ethical review board in Stockholm, Sweden, approved the study (DNR 2010/1014-31/1).

Twenty-eight participants with hip OA completed the pre- and postoperative motion analysis and were included in the study (Table 1). Reasons for failure to follow-up and/or exclusion were THA in the contralateral limb (*n* = 3), not able to perform the 5STS without the use of arms to push of seat (*n* = 2), and occluded markers during the test (*n* = 7). In the control group, four individuals were excluded due to occluded markers. The excluded individuals with hip OA (*n* = 12) did not differ statistically from the included individuals with hip OA (*n* = 28), this with respect to age, weight, height, BMI, duration of years with symptomatic hip OA, gender distribution, and time to perform the 5STS.

**Table 1:** Baseline characteristics of individuals with hip osteoarthritis and a healthy control group included in the study

	Hip osteoarthritis, ***n*** = 28	Control group, ***n*** = 21	Statistical difference between groups, *p*
**Female**, *n* (%)	18 (64)	13 (62)	0.864
**Age**, years, mean (SD)	66.0 (9.0)	66.2 (9.7)	0.948
**Heigh**t (cm), mean (SD)	170 (9.0)	171 (8.0)	0.743
**Weight** (kg), mean (SD)	76.3 (16.0)	72.6 (12.5)	0.386
**BMI** (m2/kg), mean (SD)	26.2 (4.2)	24.8 (3.0)	0.184
**Symptom duration**, years, median (range)	3.5 (0.5–12.5)	–	
**Kellgren Lawrence grade (1–4b)**			
**1–2**	–	–	
**3a,***n* (%)	2 (7)	–	
**3b,***n* (%)	7 (25)	–	
**4a,***n* (%)	10 (36)	–	
**4b,***n* (%)	9 (32)	–	
**Hip osteoarthritis outcome score (0–100)**			
**Pain**	44 (11)	98 (4)	**< 0.001**
**Symptoms**	40 (16)	96 (5)	**< 0.001**
**ADL**	49 (14)	97 (7)	**< 0.001**
**Sport and recreation**	29 (18)	96 (10)	**< 0.001**
**Hip-related quality of life**	27 (13)	95 (9)	**< 0.001**

*ADL* activities of daily living, *SD* standard deviation

### Three-dimensional motion analysis

Motion analyses were conducted at the Motion Analysis Laboratory at Karolinska University Hospital using an 8 camera system (Vicon Motion Systems Ltd, Oxford, UK) and a conventional biomechanical model, the Plug-In-Gait full-body (Appendix 1 [25]).The Plug-In Gait model consists of 35 retro-reflective markers and was used to calculate the CoM [26]. Individuals with OA were evaluated twice: once within 1 month prior to THA and again 1 year (12.2 ± 1.1 months) postoperatively.

### The five times sit-to-stand test

A bench with a seat height of 44.5 cm, without arm or backrest, was used for all the recordings of the test (Appendix 2 [25]). Participants were instructed to rise from a seated position to a standing position as fast as possible five times consecutively, with the arms placed across their chest [10]. Time to perform the test, including all five repetitions, was recorded to a hundredth of a second using a stopwatch. All participants were asked to perform the test twice to ensure complete 3D data for all participants and to observe influence of fatigue and/or any learning effect. The fastest trial was used for further analysis. One practice repetition preceded the test. Perceived pain during the test was rated by the participant after each trial according to a visual analogue scale (VAS), which ranges across a continuum from “no pain” to “worst imaginable pain.”

### Hip Disability and Osteoarthritis Outcome Score

All participants completed the Hip Disability and Osteoarthritis Outcome Score (HOOS) [27]. HOOS is a joint-specific self-assessment questionnaires, reliable for assessing baseline function and change over time in individuals with hip OA [27]. The questionnaire is divided into five subscales and each subscale generates a score ranging from 0 to 100 where 0 represent “worst” and 100 “best.”

### Radiographic severity of hip osteoarthritis

Preoperative radiographs were collected according to standard procedures at each hospital. Radiographic classification of OA was carried out by two experienced orthopedic surgeons according to the modified Kellgren-Lawrence grade ranging from 1 to 4b [28].

### Hip arthroplasty and postoperative rehabilitation

Five senior orthopedic surgeons performed the THA surgeries using an anterolateral approach. At both hospitals, postoperative regimens allowed full weight bearing from start and no movement restrictions. After surgery, all individuals with THA were encouraged to use an appropriate walking aid to support a normal gait pattern and avoid limping. Postoperative rehabilitation was performed according to the standard practice at each hospital and, thereafter, in a primary care setting of the patient’s choice. Anecdotally, reported by individuals with THA at the 1-year follow-up, the time period they used a walking aid lasted for 2 months, and the rehabilitation period lasted for a median duration of 3 (range 0–12) months.

### Data reduction

**Fig. 1:**
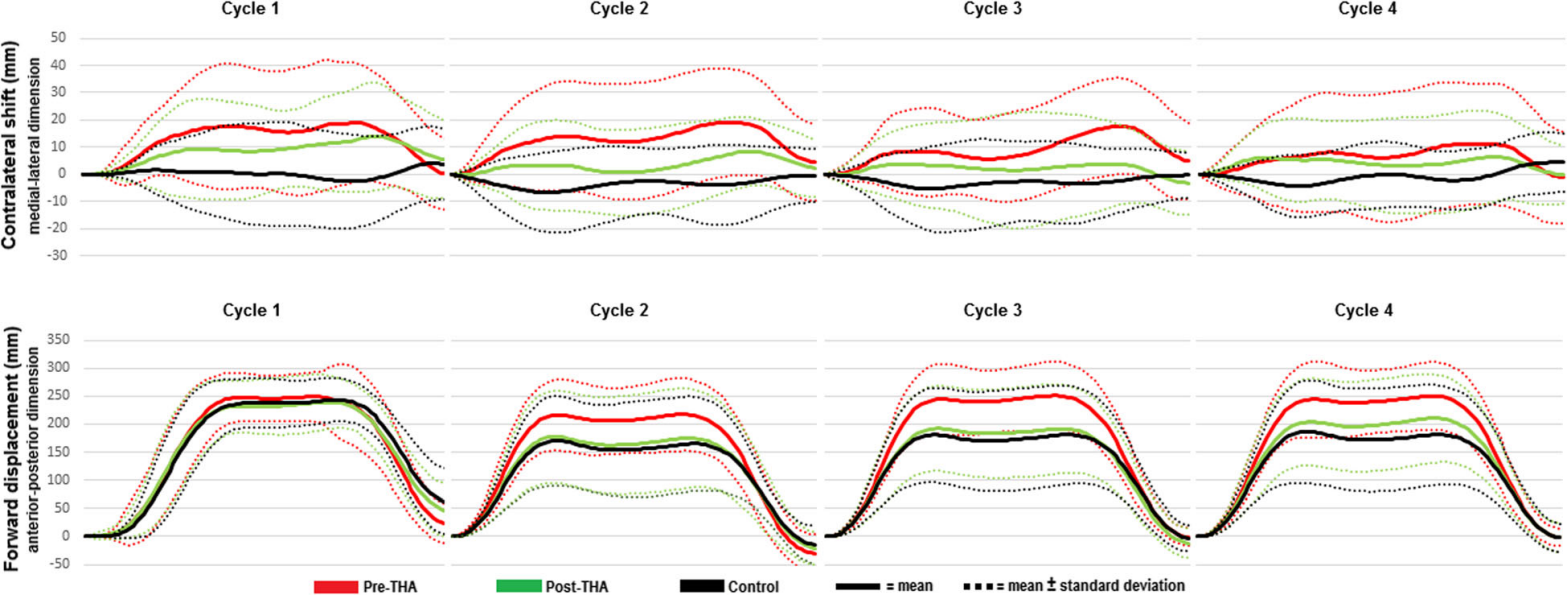
The center of mass trajectory during the five times sit-to-stand test in individuals with hip osteoarthritis within 1 month prior to total hip arthroplasty (THA), 1 year postoperatively, and in a healthy control group. The contralateral shift is observed in the medial-lateral direction and the forward displacement in the anterior posterior direction

Each 5STS was analyzed, and events were defined, using Nexus Software (Nexus 2.5. VICON Oxford Metrics, Oxford, UK). For each trial, six events were defined: the start of the first cycle and the end of each subsequent cycle. The beginning of each cycle, and subsequently the end of the previous cycle, was defined as the position when the trunk was neutral in term of its position to the global vertical axis in the lab. As the last cycle only included the sit-to-stand transition, it was not considered a full cycle and was excluded, leaving four full cycles to be included in the evaluation. Motion analysis data were exported to Matlab software (The Mathworks Inc., Natick, M.A., 2010), where each cycle was scaled to 100 data points in the medial-lateral and anterior-posterior dimension to enable comparisons between trials performed at different speeds. The mean trajectories with standard deviations of the CoM in both directions, from the cycles on a time scale of 100, were plotted for visual analysis purposes (Fig. 1). Data of patients with hip OA in the medial-lateral dimension were converted according to their affected and non-affected limb. The area under the curve (AUC) of the CoM trajectories was calculated for each cycle separately in both dimensions.

### Statistical analyses

Statistical analyses were performed using IBM SPSS Statistics version 23 (SPSS Inc., Chicago, IL). The Shapiro-Wilk test and Q-Q plots were used to evaluate the distribution of data. A *p* value below 0.05 was considered statistically significant. To evaluate the differences between pre- and postoperative baseline characteristics and time to perform the 5STS, paired sample *t* tests were used. Comparisons between individuals with hip OA (pre- and postoperative) and the control group were evaluated using independent samples *t* tests. Differences in VAS pain during 5STS pre- and postoperative were examined using Wilcoxon signed-rank test. To evaluate the difference between the AUC pre- and postoperative, a two-way repeated measurements ANOVA was performed. This included a within-factor of session (pre- vs. postoperative), a within-factor of cycle (1–4), and a session × cycle interaction. The session × cycle interaction refers to the statistical test of whether the changes from cycle to cycle differ by session. Two separate two-way mixed design ANOVAs were used to investigate the differences in AUC between individuals with hip OA (pre- and postoperative) and the control group. This included a between factor of group (pre-/postoperative vs. control), a within-factor of cycle (1–4), and a group × cycle interaction. In addition, if the interaction analyses showed significant differences, simple effects were evaluated using Bonferroni corrections.

## Results

There were no differences between individuals with hip OA and the control group with regard to age, height, BMI, and gender distribution (Table 1). Individuals with hip OA preformed the 5STS, including all five repetitions, significantly slower than controls. One year after surgery, time to complete the 5STS and perceived pain while performing the test was significantly reduced (Table 2). At 1 year postoperatively, time to complete the 5STS was comparable to the control group (Table 2).

**Table 2:** Differences in time to complete the five times sit-to-stand test and perceived pain when performing the test among individuals with hip osteoarthritis prior to surgery, 1 year after total hip arthroplasty, and a healthy control group

	Hip OA pre THA (*n* = 28)	Hip OA post THA (*n* = 28)	Control group (***n*** = 21)	∆ Pre THA vs. control			∆ Post THA vs. control			∆ Pre THA vs. post THA		
				∆	***p***	95% CI	∆	***p***	95% CI	∆	***p***	95% CI
**Time, seconds,** mean (SD)	15.1 (4.2)	11.5 (2.9)	10.1 (3.0)	**5.0**	**< 0.001**	**[2.8, 7.1]**	1.4	0.119	[− 0.4, 3.1]	**3.6**	**< 0.001**	**[1.8, 5.4]**
**VAS pain**, **0–100**, median (range)	21 (0–81)	0 (0–35)	–	**–**			**–**			**21**	**< 0.001**	**[− 4.9, − 1.6]**

*OA* osteoarthritis, *SD* standard deviation, *THA* total hip arthroplasty, *VAS* visual analog scale

### Individuals with hip osteoarthritis preoperative vs. controls

In the medial-lateral dimension, a significantly larger contralateral shift of the CoM was observed in cycles 1–3 in individuals with hip OA as compared to the control group (Table 3). In the anterior-posterior dimension, individuals with hip OA displayed a larger forward displacement prior to surgery in cycles 2–4, but not in cycle 1, as compared to controls (Table 3). Significant main effects for group and session, respectively, and for cycle were found in individuals with hip OA prior to THA as compared to the control group (Table 3).

**Table 3:** Results of ANOVA analyses of the area under the curve of all four cycles in each direction respectively

Direction	M/L	A/P	M/L	A/P	M/L	A/P	M/L	A/P	M/L	A/P
	**Control**				**Control vs. hip OA pre THA**					
	**Area under the curve** (mm^2^)		**Between group differences for each cycle**		**Interaction** **Group × cycle**		**Main effect of group (between group differences)**		**Main effect of cycle (within group differences)**	
	mean (SD)		*p*		*p*					
**Cycle 1**	39 (1159)	15765 (2536)	**0.003**	0.806	0.173	**< 0.001**	**< 0.001**	**0.022**	0.197	**< 0.001**
**Cycle 2**	− 326 (1065)	11271 (5521)	**< 0.001**	**0.043**						
**Cycle 3**	− 261 (1019)	12501 (6023)	**< 0.011**	**0.006**						
**Cycle 4**	− 76 (732)	12576 (6101)	0.084	**0.007**						
	**Hip OA pre THA**				**Hip OA pre THA vs. hip OA post THA**					
	**Area under the curve** (mm^2^)		**Between session differences for each cycle**		**Interaction** **Group × cycle**		**Main effect of session (between session differences)**		**Main effect of cycle (within group differences)**	
	mean (SD)		*p*		*p*					
**Cycle 1**	1238 (1430)	15578 (2692)	0.110	0.819	0.281	**0.001**	**0.013**	**0.009**	**0.016**	**< 0.001**
**Cycle 2**	1201 (1530)	14212 (4364)	**0.011**	**0.030**						
**Cycle 3**	885 (1087)	16564 (3866)	**0.019**	**0.001**						
**Cycle 4**	631 (1716)	16626 (4031)	0.528	**0.016**						
	**Hip OA post THA**				**Control vs. hip OA post THA**					
	**Area under the curve** (mm^2^)		**Between group differences for each cycle**		**Interaction** **Group × cycle**		**Main effect of group (between group differences)**		**Main effect of cycle (within group differences)**	
	mean (SD)		*p*		*p*					
**Cycle 1**	829 (1132)	15423 (3146)	**0.021**	0.685	0.761	0.381	**0.010**	0.706	0.062	**< 0.001**
**Cycle 2**	337 (1036)	11754 (6072)	**0.033**	0.780						
**Cycle 3**	201 (1291)	12910 (5320)	0.182	0.803						
**Cycle 4**	408 (1177)	14072 (5425)	0.104	0.370						

*A/P* anterior-posterior direction, *M/L* medial-lateral direction, *OA* osteoarthritis, *SD* standard deviation, *THA* total hip arthroplasty

### Individuals with total hip arthroplasty vs. controls

One year after surgery, the contralateral shift was reduced as compared to preoperatively (Fig. 1). Despite the reduced AUC of the CoM trajectories after surgery, individuals with THA displayed a persistent significantly larger contralateral shift of the CoM than the control group in cycles 1 and 2 (Table 3). Postoperatively, there were no remaining differences in CoM trajectories in the anterior-posterior dimension between individuals with THA and controls (Table 3).

## Discussion

### Major findings

This study aimed to evaluate functional movement compensation in individuals with hip OA performing the 5STS, and change following THA. To this end, CoM trajectories in the medial-lateral and anterior-posterior dimensions were quantified prior to and 1 year after THA and compared to a healthy control group. Prior to surgery, individuals with hip OA displayed an increased contralateral shift of their CoM. At 1 year after surgery, perceived pain decreased substantially, and the contralateral shift was reduced. However, as compared to controls, individuals with THA still displayed a persistent increased contralateral shift.

### Differences in center of mass trajectories preoperative vs. control

Prior to surgery, individuals with hip OA presented with an increased contralateral shift of the CoM towards the non-affected side compared to controls. This may be symptomatic of a strategy to unload the affected limb to reduce pain [29]. Individuals with hip OA displayed an increased forward displacement of the CoM during cycles 2–4 as compared to controls. However, there were no differences between individuals with hip OA and the controls during cycle 1 in the anterior-posterior dimension (Fig. 1). It appears that healthy controls are able to adapt their movement pattern, to be more efficient (i.e., just lean forward as much as needed to complete the specific task) following the first cycle, while individuals with OA do not display the capacity to do so [15].

### Differences in center of mass trajectories preoperative vs. postoperative

Confirming the hypothesis, the contralateral shift was reduced 1 year after surgery, alongside reductions in perceived pain measured using a VAS and time to complete the test. Although the contralateral shift of the CoM decreased after surgery, individuals with THA still displayed a significant asymmetry in the medial-lateral dimension as compared to healthy controls. The asymmetric movement pattern following THA may not solely be driven by pain [29], as the majority of individuals with THA did not report any perceived pain while performing the 5STS postoperatively. Instead, the persistent unloading of the affected limb may be maintained as a result of a compensatory movement pattern that has developed over several years of living with OA.

### Clinical implications

At 1 year after joint replacement, time to complete the 5STS did not differ between individuals with THA as compared to controls. These findings indicate that *time* to perform the 5STS is not able to detect differences in performance and cannot capture the underlying biomechanical deficits. Performance-based tests are valuable for collecting information on what individuals *can* do. However, what performance-based tests fail to answer, is *how* they do it. The “*how*” is important since asymmetrical loading over a long period of time may contribute to reduced muscle strength of the affected limb by disuse [29]. In the present study, the asymmetrical movement pattern, represented by an increased contralateral CoM shift, was evident in cycles 1 and 2 post THA. In the subsequent cycles (3 and 4) the contralateral shift reduced, and the movement was performed more efficiently, loading limbs more equally. In a real-world perspective, it is important to acknowledge the compensatory movement occurring in the beginning of the test, as the sit-to-stand transition usually only is performed once, and not several times consecutively.

Caplan et al. reported a persistent weight shift towards the non-affected side during a sit-to-stand test at 3 months after THA; however, they found symmetric limb loading at 1 year after THA [12]. In line with results of the present study, Talis et al. reported persistent asymmetric loading after a mean of 29 months after THA [22]. Regardless of the specific cause, or the most dominating contributor to asymmetric limb loading in individuals reporting little or no pain, these findings have implications for clinical rehabilitation. Focus should be directed towards normalizing loading of the limbs during not only walking, but also more strenuous tasks such as rising from a chair. Eitzen et al. express that a necessity for developing specific treatment interventions is to identify functional movement compensations and subsequent alterations in joint loading at an early stage of the disease [13]. We believe that it is equally important that the measures used to detect functional movement compensations also are able to follow change over time and after treatment interventions. Quantification of the CoM during the 5STS appears to enable detection of functional movement compensations among individuals with hip as well as knee OA [15] and seems to be responsive to change following joint replacement surgery. Even though 3D motion analysis is not feasible to conduct in clinical practice, findings from the present study are possible to implement in an everyday clinical setting. Physiotherapists, who are trained to observe and analyze functional movement, may integrate visual evaluation of 5STS performance, in addition to time taken for patients to conduct the test. Furthermore, interventions increasing patient awareness with regard to symmetrical limb loading during all activities of daily living are warranted [30].

### Limitations and strengths

The current study is not without limitations, and these should be acknowledged. First, the included study participants with hip OA reported unilateral hip pain prior to surgery and had no comorbidities. Consequently, this group of participants was relatively healthy and may not be representative for all individuals with hip OA, which makes the results a little less generalizable. Secondly, the same bench, with a fixed seat height, was used for all study participants, at all test sessions. Previous research has shown that seat height influences the outcome of STS movement as it changes the biomechanical demands needed to rise from a seated position. A higher seat height requires lower moments to be produced in the hip and knee joints, whereas a lower seat height makes the sit-to-stand movement more demanding as larger moments are required [31]. Consequently, taller individuals need to produce larger joint moments to perform the test. However, there was no significant difference in height between individuals with hip OA and the control group. In addition, the height of the bench used within this study corresponds to the height of chairs and benches out in the real world. The strengths of the present study are the use of a prospective design, the use of an age- and gender-matched control group, and the size of the study cohort, which in this context may be considered relatively large for this type of study.

### Future perspectives

All THA surgeries performed in this study cohort were done using an anterolateral approach. A recent study revealed differences in hip abductor strength at 6- and 12-month follow-ups between patients operated via three different surgical approaches [32]. Patients operated via a direct lateral approach demonstrated significantly lower hip abduction strength in the operated leg as compared to patients operated via an anterior approach or a posterior approach [32]. Consequently, it is possible that surgical approach also could have an effect on functional movement patterns, such as performance on the 5STS: however, to determine the size of its impact, future research on this specific topic is needed.

## Conclusions

Quantification of CoM trajectories in individuals with hip OA performing the 5STS appears to be able to detect functional movement compensations and to be responsive to change following THA. Prior to surgery, individuals with hip OA displayed an increased contralateral shift of their CoM, indicating a strategy to reduce pain and unload the affected limb. At 1 year after surgery, perceived pain decreased substantially, and the contralateral shift was reduced. However, as compared to controls, individuals with THA still displayed a persistent increased contralateral shift. This finding has implications for postoperative rehabilitation, where more focus must be directed towards restoring symmetrical movement patterns during demanding activities such as rising from a chair and interventions increasing patients’ awareness with regard to symmetrical movement strategies.

## Supplementary information

**Additional file 1:.** Appendix 1. Marker placement during three-dimensional motion analysis according to the Plug-In-Gait model [25].

**Additional file 2:.** Appendix 2. Schematic illustration of the set-up including evaluated directions of the center of mass displacement during the five times sit-to-stand test [25].

## Data Availability

The datasets that are used and analyzed for the present study are available from the corresponding author on reasonable request.

## Abbreviations

AUC: Area under the curve
BMI: Body mass index
CoM: Center of mass
OA: Osteoarthritis
THA: Total hip arthroplasty
3D: Three-dimensional
5STS: Five times sit-to-stand test
