# Supplementary figures and images for: Functional movement compensations persist in individuals with hip osteoarthritis performing the five times sit-to-stand test 1 year after total hip arthroplasty
Source: J Orthop Surg Res. 2020 Apr 16;15:151. doi: 10.1186/s13018-020-01663-0 (PMC7164189; doi:10.1186/s13018-020-01663-0)

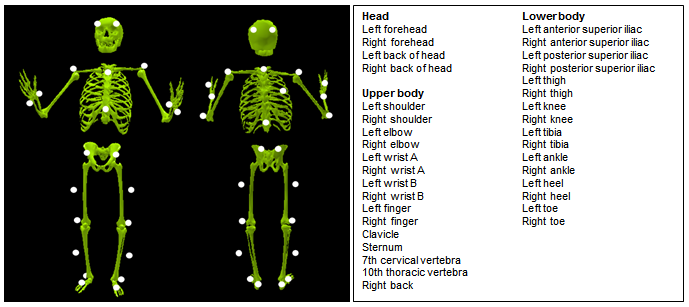

Supplement: Supplementary file 1 — Additional file 1:. Appendix 1. Marker placement during three-dimensional motion analysis according to the Plug-In-Gait model [25]. [file 13018_2020_1663_MOESM1_ESM.tif]

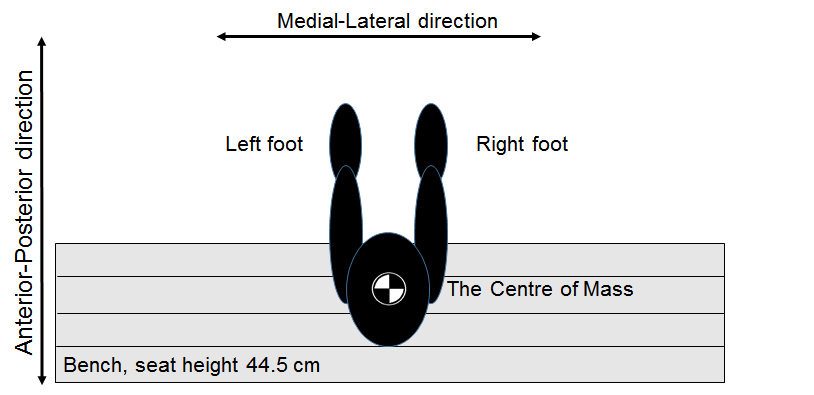

Supplement: Supplementary file 2 — Additional file 2:. Appendix 2. Schematic illustration of the set-up including evaluated directions of the center of mass displacement during the five times sit-to-stand test [25]. [file 13018_2020_1663_MOESM2_ESM.tif]
